# Vitamin C alleviates acute enterocolitis in *Campylobacter jejuni* infected mice

**DOI:** 10.1038/s41598-020-59890-8

**Published:** 2020-02-19

**Authors:** Soraya Mousavi, Ulrike Escher, Elisa Thunhorst, Sophie Kittler, Corinna Kehrenberg, Stefan Bereswill, Markus M. Heimesaat

**Affiliations:** 1 Institute for Microbiology, Infectious Diseases and Immunology, Gastrointestinal Microbiology Research Group, Charité – Universitätsmedizin Berlin, corporate member of Freie Universität Berlin, Humboldt-Universität zu Berlin, and Berlin Institute of Health, Berlin, Germany; 2 Institute for Food Quality and Food Safety, University of Veterinary Medicine Hannover, Hannover, Germany; 3 Institute for Veterinary Food Science, Justus-Liebig-University, Giessen, Germany

**Keywords:** Immunology, Antimicrobial responses, Bacterial infection, Acute inflammation, Pathogens

## Abstract

Human foodborne infections with the zoonotic pathogen *Campylobacter jejuni* are on the rise and constitute a significant socioeconomic burden worldwide. The health-beneficial, particularly anti-inflammatory effects of vitamin C (ascorbate) are well known. In our preclinical intervention study, we assessed potential anti-pathogenic and immunomodulatory effects of ascorbate in *C. jejuni*-infected secondary abiotic IL-10^−/−^ mice developing acute campylobacteriosis similar to humans. Starting 4 days prior peroral *C. jejuni*-infection, mice received synthetic ascorbate via the drinking water until the end of the experiment. At day 6 post-infection, ascorbate-treated mice harbored slightly lower colonic pathogen loads and suffered from less severe *C. jejuni*-induced enterocolitis as compared to placebo control animals. Ascorbate treatment did not only alleviate macroscopic sequelae of infection, but also dampened apoptotic and inflammatory immune cell responses in the intestines that were accompanied by less pronounced pro-inflammatory cytokine secretion. Remarkably, the anti-inflammatory effects of ascorbate pretreatment in *C. jejuni*-infected mice were not restricted to the intestinal tract but could also be observed in extra-intestinal compartments including liver, kidneys and lungs. In conclusion, due to the potent anti-inflammatory effects observed in the clinical murine *C. jejuni*-infection model, ascorbate constitutes a promising novel option for prophylaxis and treatment of acute campylobacteriosis.

## Introduction

*Campylobacter jejuni* are the most common cause of food-borne gastroenteritis with increasing prevalence worldwide^1,2^. In fact, human campylobacteriosis represents a socioeconomic burden given estimated disease-associated costs of approximately 2.4 billion Euro^3^. Most commonly, *C. jejuni* transfer via consumption of contaminated raw or undercooked meat and milk or the ingestion of contaminated surface water to humans^4–8^. The intestinal colonization of *C. jejuni* induces a strong inflammatory response of the innate immune system affecting both, absorptive and secretory functions of the gastrointestinal tract^1^. In fact, campylobacteriosis constitutes a classical sodium malabsorption syndrome^9^, which depending on the bacterial strain and the host immune status, results in illness of varying degree^10^. Whereas some patients remain asymptomatic or display mild symptoms, others develop fever, abdominal pain and watery diarrhea, or suffer from acute campylobacteriosis characterized by severe enterocolitis with inflammatory, bloody diarrhea^1,11^. In the majority of events, the disease is self-limited and treated symptomatically, whereas patients with immunosuppressive comorbidities require antibiotic treatment^11,12^. However, in few instances, post-infectious sequelae including Guillain-Barré syndrome, Miller Fisher syndrome, reactive arthritis and chronic inflammatory conditions of the intestinal tract might develop with a latent period of weeks or longer^1,13^.

Even though human campylobacteriosis is becoming increasingly important, the distinct cellular and molecular mechanisms of host-pathogen interactions are limited. Clinical investigations in human patients disclosed that severe courses of *Campylobacter* infection and post-infectious morbidities (e.g., Guillain-Barré syndrome) are induced by the Gram-negative bacterial cell wall constituent lipooligosaccharide (LOS), especially sialylated LOS, which leads to hyper-activating of immune response^14^. Further RNA sequencing studies in human volunteers confirmed the major role of LOS-induced toll-like receptor (TLR) -4 signaling pathways in the induction of acute campylobacteriosis^9^. These findings support the view that campylobacteriosis results from a LOS-induced, TLR-4 mediated hyperergic innate immune response, which is similar to the inflammatory events induced by other LOS producing pathogens like *Neisseria meningitidis* and *N. gonorrhoeae*. However, *in vivo* trials have been hampered by the limited availability of experimental models. Mice show a strong physiological colonization resistance against invading microorganisms due to the mouse specific gut microbiota composition and are therefore protected from infection with enteropathogenic bacteria such as *C. jejuni*^15–17^. Furthermore, when compared to human, mice have been shown to be about 10,000-fold more resistant against LOS and lipopolysaccharide (LPS) expressed by Gram-negative bacteria^18–20^. We could recently show that a depletion of the gastrointestinal microbiota upon broad-spectrum antibiotic application in IL-10^−/−^ mice facilitates intestinal *C. jejuni* colonization resulting in the development of key symptoms of acute human campylobacteriosis including wasting and bloody diarrhea within several days post-infection^21^. The main reasons for these severe *C. jejuni* induced immunopathological responses mounting in acute ulcerative enterocolitis are (i.) the abrogation of colonization resistance following microbiota depletion and (ii.) the lack of IL-10 enhancing susceptibility of mice to *C. jejuni* LOS^21^. In consequence, secondary abiotic IL-10^−/−^ mice challenged with *C. jejuni* show strong intestinal and extra-intestinal immune responses via LOS–induced TLR-4 signaling^21–30^. Most importantly, the major role of LOS-induced intestinal immunopathology during campylobacteriosis was independently confirmed in elegant infection experiments with microbiota depleted SIGGR^−/−^ mice developing campylobacteriosis similar to secondary abiotic IL-10 deficient mice. In contrast to the latter, the SIGGR^−/−^ mice rendered sensitive to LOS due to the lack of a central inhibitor of cellular LPS/LOS-induced signaling pathways^31^.

In the 1920s, vitamin C was first isolated by the Hungarian Nobel laureate Albert Szent Györgyi on track to unravel the options for treatment and prophylaxis of morbidities such as scurvy caused by deficiency of this (for humans essential) vitamin^32,33^. Patients suffering from scurvy exhibit poor wound healing due to weakening of collagenous structures and compromised immune cell functions^34^ and are therefore highly susceptible to infections^32,35^. Ascorbate, the biologically active form of vitamin C, exerts a strong reductive potential and acts as a potent antioxidant that can be reversibly oxidized to dehydroascorbic acid^33,36^. Due to these characteristics, ascorbate is involved in several pivotal host defenses including immune regulatory pathways^35^. This is further underlined by the fact that both, innate and adaptive immune cells such as neutrophils, monocytes and lymphocytes, respectively, can accumulate ascorbate against a concentration gradient and exhibit intracellular concentrations that are up to 100 times higher than in plasma^36–38^. The presence of ascorbate in cells and plasma protects from oxidative stress. It is known that during phagocytosis, human granulocytes release hydrogen peroxide (H_2_O_2_) into the extracellular medium subsequently causing oxidative stress^39^ and cell damage by lipid peroxidation and alteration of protein and nucleic acid structure^40^. Interestingly, due to this potent antioxidant property, ascorbate has been shown to neutralize H_2_O_2_ and to reduce the H_2_O_2_-induced apoptosis in periodontal tissues^41^. Moreover, endotoxin-induced oxidative stress due to reactive oxygen species (ROS) is associated with high cell mortality^42^. Increasing intracellular ascorbate concentrations, however, decrease ROS levels, thereby counteracting cell mortality^43^.

Ascorbate has been shown to exert antimicrobial effects against distinct bacterial species such as *Mycobacterium tuberculosis*^44–47^, *Staphylococcus aureus, Escherichia coli*^48^, *Helicobacter pylori*^49,50^ and *Salmonella* species^51^ *in vitro*. Beside direct effects of ascorbate due to its low pH, for instance, reducing cell viability, it is also known that ascorbate exerts indirect bactericidal effects in the presence of metal ions or oxygen^52^. However, bacterial biofilm protects the cells against external influences and causes increased tolerance to antibiotic compounds^53^. Interestingly, ascorbate disrupts bacterial biofilm formation by inhibiting production of extracellular polymeric substances in *Bacillus subtilis*^54^ and methicillin-resistant *S. aureus* (MRSA)^55^ and pyocyanin production in *Pseudomonas aeruginosa*^56^. Thus, vitamin C (alone or in combination with antibiotics) constitutes a promising treatment option to destabilize bacterial biofilms.

Studies in the early 1980s revealed that growth of enteropathogens such as *C. jejuni* could be inhibited by ascorbate at low concentrations^52,57,58^. The authors have elegantly shown that the antimicrobial activity of ascorbate against *C. jejuni* is not due to lowered pH, but rather depends on the oxidation of ascorbate to dehydroascorbate and other products. However, the antimicrobial effects of ascorbate against *C. jejuni* have not been examined further and the exact mechanisms underlying the ascorbate-mediated toxicity against *C. jejuni* are still not known in detail. Moreover, data regarding potential immunomodulatory effects of ascorbate in *C. jejuni* infection are completely missing. This prompted us to perform for the first time a preclinical ascorbate intervention study applying our well-established murine clinical model of acute campylobacteriosis. Therefore, microbiota depleted IL-10^−/−^ mice were subjected to synthetic ascorbate application via the drinking water, perorally infected with *C. jejuni* and surveyed for gastrointestinal pathogen loads, clinical outcome and intestinal as well as extra-intestinal immune responses during campylobacteriosis.

## Results

### Ascorbate treatment and gastrointestinal pathogen loads in *C. jejuni* infected secondary abiotic IL-10^−/−^ mice

We first assessed antimicrobial effects of synthetic ascorbate against *C. jejuni in vitro*. Studies on minimal inhibitory concentrations (MICs) of 20 different *C. jejuni* isolates revealed a MIC_90_ value of 2818 mg/L (16 mM) with MICs ranging between 352 and 2818 mg/L (Supplementary Fig. S1). A MIC of 1409 mg/L (8 mM) was determined for the *C. jejuni* infection strain 81–176.

**Figure 1:**
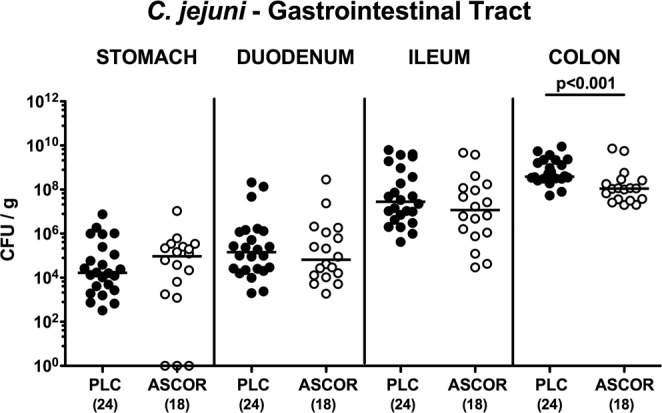
Gastrointestinal pathogen loads following ascorbate treatment of *C. jejuni* infected secondary abiotic IL-10^−/−^ mice. Starting four days before peroral *C. jejuni* infection on days 0 and 1, secondary abiotic IL-10^−/−^ mice were treated with synthetic ascorbate (ASCOR; open circles) or placebo (PLC; filled circles) via the drinking water. At day 6 post-infection, *C. jejuni* were isolated from distinct luminal parts of the gastrointestinal tract by culture and pathogenic loads expressed as colony forming units per gram (CFU/g). Medians, significance levels (p-values) assessed by the Mann-Whitney U test (for pairwise comparisons of PLC vs ASCOR in respective gastrointestinal compartment) and numbers of analyzed animals (in parentheses) are indicated. Data were pooled from four independent experiments.

Secondary abiotic IL-10^−/−^ mice were subjected to synthetic ascorbate treatment via the drinking water starting four days prior *C. jejuni* infection and lasting until necropsy. The concentration of the applied ascorbate solution was 5 g/L and hence, 3.56 times the MIC of the *C. jejuni* infection strain 81–176. On days 0 and 1, mice were then perorally challenged with 10^9^ viable *C. jejuni* bacteria by gavage and surveyed until day 6 post-infection (p.i.). Bacterial culture analysis of faecal samples over time revealed that ascorbate treatment did not affect intestinal colonization properties of the applied *C. jejuni* strain as indicated by comparable median loads of approximately 10^9^ colony forming units per gram (CFU/g) derived from ascorbate or placebo (PLC) treated mice (n.s.; Supplementary Fig. S2). At day 6 p.i., colonic *C. jejuni* loads were slightly lower in ascorbate as compared to placebo treated mice (i.e., less than one log order of magnitude; p < 0.001; Fig. 1), whereas pathogen numbers were comparable in more proximal parts of the gastrointestinal tract including stomach, duodenum and ileum (n.s.; Fig. 1). Hence, ascorbate does only marginally affect intestinal *C. jejuni* colonization *in vivo*.

### Ascorbate treatment ameliorates the clinical outcome of campylobacteriosis in *C. jejuni* infected secondary abiotic IL-10^−/−^ mice

**Figure 2:**
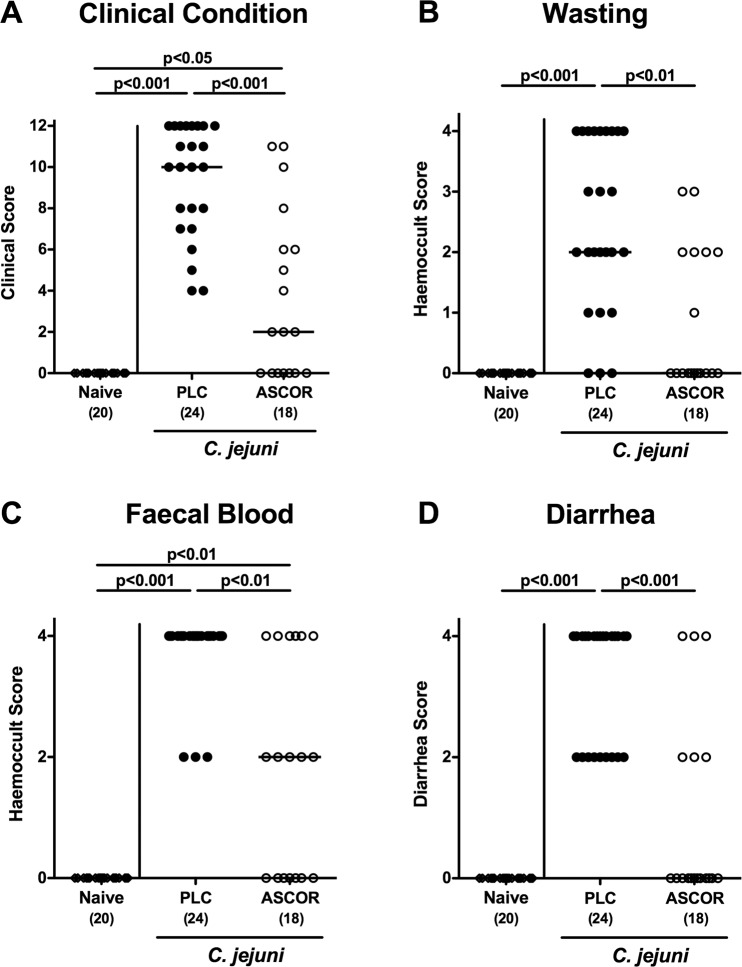
Macroscopic aspects of ascorbate treated mice following *C. jejuni* infection. Starting four days before peroral *C. jejuni* infection on days 0 and 1, secondary abiotic IL-10^−/−^ mice were treated with synthetic ascorbate (ASCOR; open circles) or placebo (PLC; filled circles) via the drinking water. Applying a standardized clinical scoring system (see methods), **(A)** overall clinical outcome, **(B)** wasting, **(C)** abundance of faecal blood and **(D)** stool consistency were quantitatively surveyed at day 6 post-infection. Naive mice (open diamonds) served as uninfected controls. Medians, significance levels (p-values) assessed by the Kruskal Wallis test followed by Dunns correction and numbers of analyzed animals (in parentheses) are indicated. Data were pooled from four independent experiments.

A kinetic survey of the clinical conditions of ascorbate versus PLC treated mice revealed that as early as day 5 p.i., the former suffered less distinctly from *C. jejuni* infection as compared to the latter (Supplementary Fig. S3). Upon necropsy (i.e., at day 6 p.i.), placebo treated mice were suffering from wasting and severe bloody diarrhea indicative for acute enterocolitis, whereas ascorbate treatment resulted in significantly reduced *C. jejuni* induced symptoms (Fig. 2A; Supplementary Fig. S3), particularly in less severe wasting, less pronounced diarrhea, less frequent abundance of blood in faecal samples and in better overall clinical appearance (p < 0.05–0.001 vs PLC; Fig. 2). Remarkably, almost 40% of infected mice from the ascorbate cohort were clinically uncompromised as indicated by cumulative clinical scores of 0 in 7 out of 18 cases (p < 0.001 vs PLC; Fig. 2A; Supplementary Fig. S3). Hence, ascorbate treatment alleviates clinical symptoms of campylobacteriosis.

### Ascorbate reduces apoptosis in the colon of *C. jejuni* infected secondary abiotic IL-10^−/−^ mice

**Figure 3:**
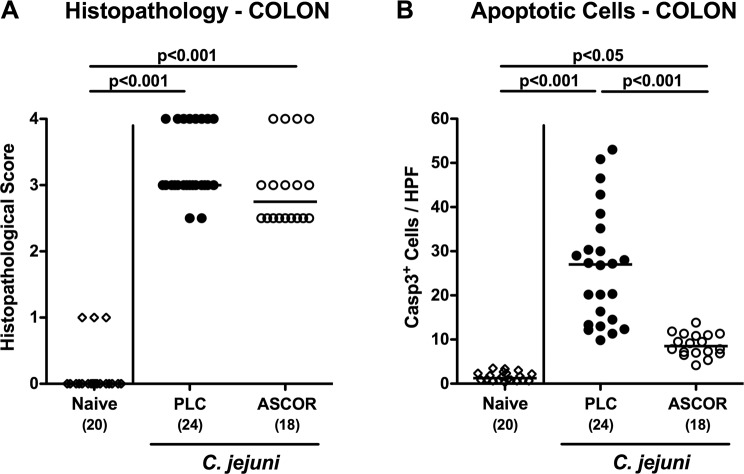
Histopathological and apoptotic cell responses in colonic epithelia of ascorbate treated mice following *C. jejuni* infection. Starting four days before peroral *C. jejuni* infection on days 0 and 1, secondary abiotic IL-10^−/−^ mice were treated with synthetic ascorbate (ASCOR; open circles) or placebo (PLC; filled circles) via the drinking water. **(A)** Histopathological changes were quantitated in hematoxylin and eosin stained colonic paraffin sections applying a standardized scoring system as described in methods. **(B)** The average numbers of apoptotic (positive for caspase3, Casp3^+^) colonic epithelial cells from six high power fields (HPF, 400x magnification) per mouse were assessed microscopically in immunohistochemically stained large intestinal paraffin sections at day 6 post-infection. Naive mice (open diamonds) served as uninfected and untreated controls. Medians, significance levels (p-values) assessed by the one-way ANOVA test followed by Tukey correction and numbers of analyzed animals (in parentheses) are indicated. Data were pooled from four independent experiments.

We next quantitatively assessed pathogen-induced histopathological changes in hematoxylin and eosin (H&E) stained colonic paraffin sections applying a standardized histopathological scoring system^59^. At day 6 p.i., ascorbate treated mice displayed a trend towards slightly lower histopathological scores as compared to PLC control animal (n.s.; Fig. 3A).

Since apoptosis is regarded as a reliable parameter for the histopathological grading of intestinal inflammation^15^, we further quantitatively determined apoptotic colonic epithelial cell numbers following *in situ* immunohistochemical staining of colonic paraffin sections with a caspase3 antibody. In fact, *C. jejuni* infection was associated with multifold increases in colonic apoptotic epithelial cells at day 6 p.i. (p < 0.001 vs naive, whereas this increase was far less pronounced in ascorbate as compared to PLC treated mice (p < 0.001; Fig. 3B; Supplementary Fig. S4). Hence, ascorbate treatment decreases *C. jejuni* induced apoptotic responses in colonic epithelial cells.

### Less distinct pro-inflammatory immune cell responses in the colon following ascorbate treatment of *C. jejuni* infected secondary abiotic IL-10^−/−^ mice

**Figure 4:**
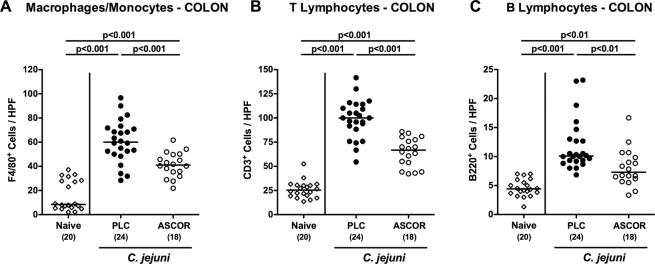
Colonic immune cell responses in ascorbate treated mice following *C. jejuni* infection. Starting four days before peroral *C. jejuni* infection on days 0 and 1, secondary abiotic IL-10^−/−^ mice were treated with synthetic ascorbate (ASCOR; open circles) or placebo (PLC; filled circles) via the drinking water. The average numbers of **(A)** macrophages and monocytes (positive for F4/80), **(B)** T lymphocytes (positive for CD3), and **(C)** B lymphocytes (positive for B220) in the colonic mucosa and lamina propria from six high power fields (HPF, 400x magnification) per mouse were assessed microscopically in immunohistochemically stained large intestinal paraffin sections at day 6 post-infection. Naive mice (open diamonds) served as uninfected and untreated controls. Medians, significance levels (p-values) assessed by the one-way ANOVA test followed by Tukey correction and numbers of analyzed animals (in parentheses) are indicated. Data were pooled from four independent experiments.

We next assessed large intestinal immune cell responses following ascorbate treatment of *C. jejuni* infected mice. *C. jejuni* infection was associated with marked increases of both, innate immune cell populations such as macrophages and monocytes (p < 0.001; Fig. 4A; Supplementary Fig. S5a) and adaptive immune cell subsets including T and B lymphocytes (p < 0.01–0.001; Fig. 4B,C; Supplementary Fig. S5b,c). At day 6 p.i., however, ascorbate treated mice displayed lower numbers of macrophages/monocytes as well as of T and B lymphocytes in their colonic mucosa and lamina propria as compared to infected PLC control animals (p < 0.01–0.001; Fig. 4; Supplementary Fig. S5). Hence, ascorbate treatment does not only alleviate macroscopic disease and colonic epithelial apoptosis following *C. jejuni* infection, but also dampens immune cell responses in the infected large intestines.

### Reduced pro-inflammatory mediator secretion in the intestinal tract following ascorbate treatment of *C. jejuni* infected secondary abiotic IL-10^−/−^ mice

**Figure 5:**
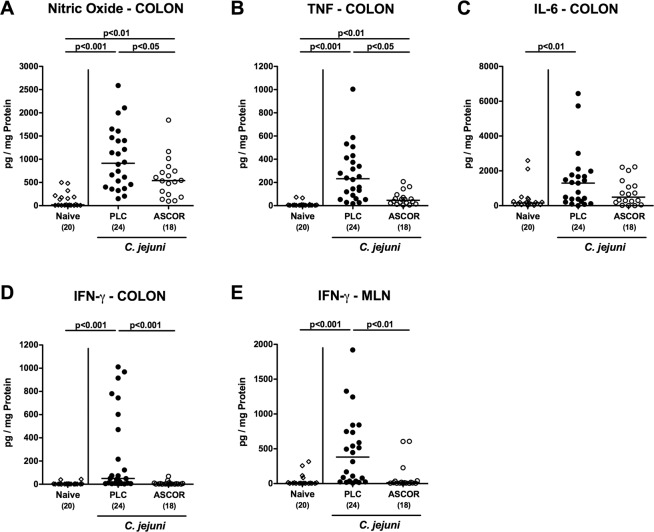
Intestinal inflammatory mediator responses in ascorbate treated mice following *C. jejuni* infection. Starting four days before peroral *C. jejuni* infection on days 0 and 1, secondary abiotic IL-10^−/−^ mice were treated with synthetic ascorbate (ASCOR; open circles) or placebo (PLC; filled circles) via the drinking water. At day 6 post-infection. pro-inflammatory mediators including **(A)** nitric oxide, **(B)** TNF, **(C)** IL-6 and **(D)** IFN-γ were measured in supernatants of *ex vivo* biopsies derived from the colon as well as (**E**) IFN-γ concentrations assessed in mesenteric lymph nodes (MLN). Naive mice served as negative controls (open diamonds). Medians, significance levels (p-values) assessed by the Kruskall Wallis test followed by Dunns correction and numbers of analyzed animals (in parentheses) are indicated. Data were pooled from four independent experiments.

We further surveyed pro-inflammatory mediator responses in the intestines of ascorbate versus PLC treated *C. jejuni* infected mice. Six days following *C. jejuni* infection elevated nitric oxide (NO) and tumor necrosis factor (TNF) concentrations could be measured in colonic *ex vivo* biopsies (p < 0.01–0.001 vs naive; Fig. 5A,B), whereas ascorbate treated mice displayed lower mediator levels in their large intestines as compared to PLC control animals (p < 0.05; Fig. 5A,B). Remarkably, elevated IL-6 and interferon-γ (IFN-γ) concentrations were determined in colonic *ex vivo* biopsies at day 6 p.i. of PLC (p < 0.01 and p < 0.001 vs naive, respectively), but not ascorbate treated mice (Fig. 5C,D), which also held true for IFN-γ levels measured in mesenteric lymph nodes (MLN) at day 6 p.i. (p < 0.001; Fig. 5E). Hence, ascorbate treatment dampens pro-inflammatory mediator responses in the large intestines of *C. jejuni* infected mice.

### Amelioration of inflammatory responses in extra-intestinal compartments following ascorbate treatment of *C. jejuni* infected secondary abiotic IL-10^−/−^ mice

**Figure 6:**
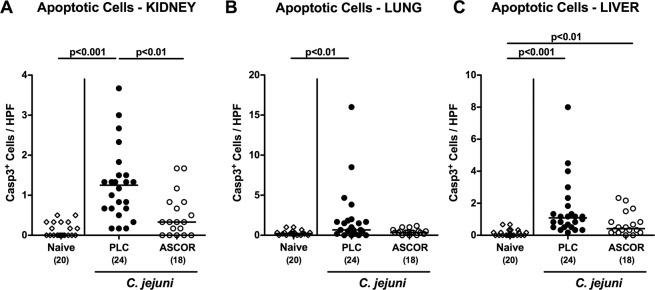
Extra-intestinal apoptosis in ascorbate treated mice following *C. jejuni* infection. Starting four days before peroral *C. jejuni* infection on days 0 and 1, secondary abiotic IL-10^−/−^ mice were treated with synthetic ascorbate (ASCOR; open circles) or placebo (PLC; filled circles) via the drinking water. At day 6 post-infection, the average numbers of apoptotic cells (positive for caspase-3, Casp3^+^) from six high power fields (HPF, 400x magnification) per mouse were assessed microscopically in immunohistochemically stained paraffin sections derived from **(A)** liver, **(B)** kidneys and **(C)** lungs. Naive mice served as uninfected and untreated controls (open diamonds). Medians, significance levels (p-values) assessed by the Kruskal Wallis test followed by Dunns correction and numbers of analyzed animals (in parentheses) are indicated. Data were pooled from four independent experiments.

**Figure 7:**
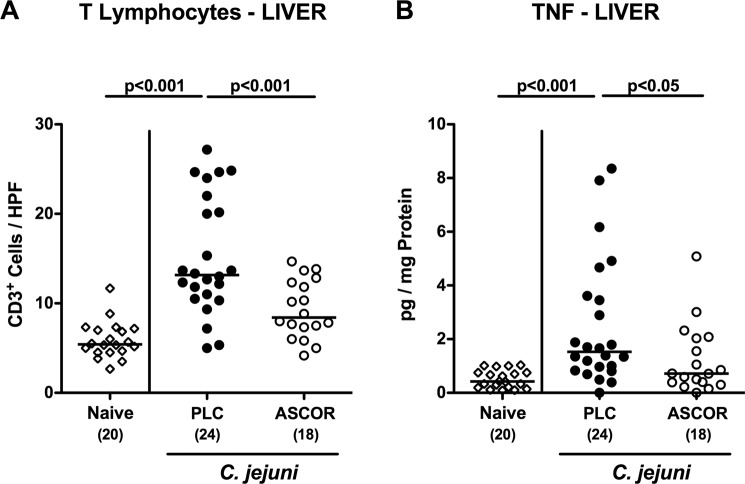
Hepatic inflammatory immune responses in ascorbate treated mice following *C. jejuni* infection. Starting four days before peroral *C. jejuni* infection on days 0 and 1, secondary abiotic IL-10^−/−^ mice were treated with synthetic ascorbate (ASCOR; open circles) or placebo (PLC; filled circles) via the drinking water. At day 6 post-infection, the average numbers of **(A)** T lymphocytes (positive for CD3) from six high power fields (HPF, 400x magnification) per mouse were assessed microscopically in immunohistochemically stained paraffin sections derived from liver *ex vivo* biopsies. Furthermore, **(B)** TNF concentrations were determined in supernatants of hepatic *ex vivo* biopsies taken at day 6 p.i. Naive mice served as negative controls (open circles). Numbers of mice (in parentheses), medians (black bars) and significance levels (p-values) determined by the one-way ANOVA test followed by Tukey correction are indicated. Data shown were pooled from three independent experiments.

We further addressed whether the ascorbate associated disease-alleviating effects could also be observed in extra-intestinal compartments. We therefore quantitatively assessed apoptotic cell responses in kidneys, lungs and liver applying *in situ* immunohistochemistry. Six days following *C. jejuni* infection, increased numbers of caspase3^+^ cells could be determined in the kidneys and lungs of PLC (p < 0.01–0.001 vs naive), but not of ascorbate treated mice (Fig. 6A,B; Supplementary Fig. S6a,b). *C. jejuni* infected mice from either cohort displayed elevated hepatic apoptotic cell numbers (p < 0.01–0.001 vs naive), but with a trend towards lower numbers following ascorbate as compared to PLC application (n.s.; Fig. 6C; Supplementary Fig. S6c). Furthermore, PLC (p < 0.001 vs naive), but not ascorbate treated mice exhibited increased numbers of CD3^+^ T lymphocytes in their livers (Fig. 7A; Supplementary Fig. S6d), that was accompanied by elevated *C. jejuni* induced hepatic TNF secretion in the former, but not the latter cohort (p < 0.05 vs PLC; Fig. 7B).

We finally surveyed whether viable pathogens had translocated from the intestinal tract to extra-intestinal including systemic tissue sites following ascorbate treatment. *C. jejuni* translocation frequencies to MLN, spleen and liver were rather comparable following either treatment regimen at day 6 p.i. (Supplementary Fig. S7), whereas viable bacteria were isolated from the kidneys and lungs of PLC treated mice in single cases only, but in none of the animals from the ascorbate cohort (Supplementary Fig. S7). Of note, all blood samples taken at day 6 p.i. were *C. jejuni*-culture negative. Hence, the disease-alleviating effects of ascorbate treatment in *C. jejuni* infected mice were not restricted to the intestinal tract but could also be observed in extra-intestinal compartments.

## Discussion

In this preclinical intervention study, we addressed for the first time potential anti-pathogenic and immunomodulatory properties of vitamin C during experimental acute campylobacteriosis in a clinical murine infection model. Following adding ascorbate to the drinking water starting four days prior *C. jejuni* infection of microbiota depleted IL-10^−/−^ mice the excessively high gastrointestinal pathogen loads (of up to 10^9^ viable bacterial cells per g faeces) within the stably infected animals could only marginally be lowered by less than one order of magnitude until day 6 p.i. This result was not surprising given that the ascorbate concentration within the drinking water exceeded approximately 3.5 times the MIC against the applied *C. jejuni* strain but is reduced by mixing with the secretory body fluids in the intestinal tract of the animals. The potent antibacterial properties of ascorbate directed against food-borne pathogens such as *C. jejuni*^52,57,58^ and *Salmonella* species^51,60,61^ have been further supported by several previous *in vitro* studies. Initially, it was hypothesized that the antimicrobial effects of vitamin C were particularly due to its pH lowering properties^62^. But this is not the case for the antimicrobial activity of ascorbate against *C. jejuni*, which depends on oxidation of ascorbate to dehydroascorbic acid and other products, as confirmed in independent studies *in vitro*^52,58^.

Despite the high gastrointestinal pathogen burdens, ascorbate treatment resulted in much better overall clinical conditions of *C. jejuni* infected mice including less severe diarrhea and less frequent abundance of faecal blood. It is noteworthy that almost 40% of mice from the ascorbate cohort were clinically uncompromised whereas mice from the placebo group suffered from full-blown campylobacteriosis as indicated by wasting and bloody diarrhea. The better macroscopic outcome upon ascorbate pretreatment of *C. jejuni* infected mice was accompanied by less apoptotic colonic epithelial cell responses. In support, *Helicobacter pylori*-induced apoptosis in human gastric epithelial cells was shown to be dampened after ascorbate treatment^63^, whereas another study revealed that intracellular accumulation of ascorbate could suppress apoptotic pathways in human monocytes *in vitro*^64^. Moreover, co-incubation with ascorbate promoted proliferative properties of human peripheral T lymphocytes^65,66^.

In addition to exerting antimicrobial effects, vitamin C has been shown to exhibit potent immunomodulatory, particularly anti-inflammatory properties both, *in vitro* and *in vivo*^67–69^. In our present study, ascorbate treatment resulted in less pronounced *C. jejuni*-induced pro-inflammatory innate as well as adaptive immune responses as indicated by lower numbers of macrophages and monocytes as well as of T and B lymphocytes, respectively, within the inflamed colonic mucosa and lamina propria that were accompanied by less intestinal secretion of pro-inflammatory mediators including nitric oxide, TNF, and IFN-γ and IL-6 at day 6 p.i. In support, ascorbate co-incubation resulted in down-regulated TNF levels in human whole blood^70^ and in splenic mouse T cell cultures^71^, whereas decreased nitric oxide, TNF, IFN-γ, and IL-6 concentrations could be determined in *Staphylococcus aureu*s infected murine peritoneal macrophages *in vitro*^40^. Furthermore, ascorbate application to healthy subjects enhanced natural killer cell activities, lymphocyte proliferation and chemotaxis^72,73^, further underlining the potent immunomodulatory, anti-inflammatory effects of vitamin C^69^.

Notably, the anti-inflammatory effects of ascorbate treatment in *C. jejuni* infected mice were not restricted to the intestinal tract, but could also be observed in extra-intestinal organs as indicated by less distinct apoptosis in liver, kidneys and lungs and lower T cell numbers and lower TNF concentrations in livers of ascorbate versus placebo treated mice. Interestingly, translocation frequencies of viable *C. jejuni* from the intestinal tract to the liver were rather comparable following either treatment regimen, whereas no viable bacteria at all could be isolated from the kidneys and lungs following ascorbate pretreatment and in single cases only in placebo control mice at day 6 p.i.

The here presented immunomodulatory properties of an externally applied vitamin during acute *C. jejuni* induced enterocolitis are further supported by our very recent study surveying the health beneficial (i.e, anti-inflammatory) effects of vitamin D in the same clinical murine campylobacteriosis model. Following pre-treatment with synthetic 25-OH cholecalciferol, secondary abiotic IL-10^−/−^ mice (i.) harbored comparably high *C. jejuni* loads in their gastrointestinal tract alike placebo controls upon peroral infection, but (ii.) suffered less frequently from diarrhea in the midst of infection, displayed (iii.) less pathogen-induced apoptotic, but (iv.) more pronounced counter-regulatory regenerative colonic cell responses that were accompanied by (v.) less distinct recruitment of both, innate und adaptive immune cells to the infected intestines, and by (vi.) less secretion of pro-inflammatory mediators in the intestinal tract (i.e, colon, ileum und MLN) as well as in the liver. Furthermore, as opposed to placebo controls (vii.) vitamin D treated mice displayed an uncompromised colonic epithelial barrier function which was accompanied by (viii.) less distinct bacterial translocation from the inflamed gut to extra-intestinal compartments in the latter as compared to the former^74^.

The pharmacokinetic properties of ascorbate in the vertebrate host are well studied^75–79^. Murine investigations on absorption, tissue distribution and retention of ascorbate^76–78^ revealed that maximum ascorbate concentrations could be measured in liver and kidneys (alike in the urine of humans^75^) approximately three hours following peroral single dose application^76^. Whereas in the lungs, adrenal glands, skin, white fat and pancreas peak levels were detectable as early as 6 hours, in the spleen increasing ascorbate concentrations could still be assessed until 24 hours following application^76^. The risk of adverse effects and intoxication upon high-dose ingestion may be considered negligible in subjects with intact renal function, given that vitamin C is water-soluble and concentrations exceeding the daily demands will be excreted via the kidneys^80^.Together, ascorbate application can be considered as safe and has been pharmaceutically approved for the treatment of gastrointestinal morbidities in humans^33,60,81^. Interestingly, several inflammatory morbidities within the gastrointestinal tract have been shown to be associated with reduced ascorbate plasma concentrations^82,83^.

In conclusion, our pre-clinical intervention study provides strong evidence for the first time that ascorbate constitutes a promising compound exerting potent anti-inflammatory and hence, disease-alleviating effects in non-self-limiting acute campylobacteriosis. Furthermore, food supplementation with ascorbate might be a useful tool to enhance host defense mechanisms in livestock animals directed against enteropathogens including *C. jejuni* thereby lowering pathogenic loads or even preventing from pathogen acquisition. Further studies are needed, however, to unravel the underlying mechanism in more detail.

## Material and Methods

### Ethical statement

*In vivo* experiments were carried out according to the European Guidelines for animal welfare (2010/63/EU) following agreement by the commission for animal experiments headed by the “Landesamt für Gesundheit und Soziales” (LaGeSo, Berlin, registration number G0172/16 and G0247/16). Animal welfare was monitored twice a day.

### Determination of minimal inhibitory concentrations of ascorbate

To determine the antimicrobial effect of synthetic ascorbate, 20 *C. jejuni* isolates including the reference strain 81–176 (used for infection of mice, see below) and the DSM 4688 strain (for quality control) were tested in three independent experiments for their minimal inhibitory concentration (MIC) by the broth microdilution and macrodilution method. Settings of inoculum density, growth medium and conditions as well as incubation time were applied following the recommendations of the Clinical and Laboratory Standards Institute (CLSI) given in the document VET01-Ed5^84^. Twofold serial dilutions ranging from 0.03–32.0 mmol/L (6–5636 µg/mL) for ascorbate were tested. Stock solutions were prepared in Mueller-Hinton broth (Oxoid, Germany) and adjusted to pH 7.3.

### Generation of secondary abiotic/gnotobiotic mice

In the identical unit of the Forschungseinrichtungen für Experimentelle Medizin (FEM, Charité - University Medicine Berlin), IL10^−/−^ mice (C57BL/6j background) were bred, raised and housed under specific pathogen free (SPF) conditions. Mice were kept under standard conditions (22–24 °C room temperature, 55 ± 15% humidity, 12 h light/12 h dark cycle) in cages including filter tops within an experimental semi-barrier (accessible only with lab coat, overshoes, caps and sterile gloves) and had free access to autoclaved standard chow (food pellets: ssniff R/M-H, V1534-300, Sniff, Soest, Germany) as well as to autoclaved drinking water (*ad libitum*).

The depletion of the murine commensal intestinal microbiota in order to abrogate the physiological colonization resistance and hence, to assure stable intestinal *C. jejuni* colonization was achieved by application of five different antibiotics to the mice^15,85^. Briefly, 3-week old mice were treated with an antibiotic cocktail containing vancomycin (500 mg/L; Cell Pharm, Germany), ciprofloxacin (200 mg/L; Bayer Vital, Germany), imipenem (250 mg/L; MSD, Germany), metronidazole (1 g/L; Fresenius, Germany), and ampicillin plus sulbactam (1 g/L; Ratiopharm, Germany) within autoclaved drinking water (*ad libitum*) over a period time of 8 weeks. Three days prior infection the antibiotic treatment was withdrawn to assure antibiotic washout.

### Treatment with synthetic ascorbate

Starting four days before *C. jejuni* infection and lasting until the end of the experiment, three-month old, sex-matched secondary abiotic IL-10^−/−^ mice (maximum of three animals per cage) were treated with ascorbate (Sigma Aldrich, München, Germany) that had been sterile-filtered and added to the autoclaved tap water (*ad libitum*) and changed every other day. For ascorbate treatment, a daily dosage of 1 g per kg body weight was calculated^86^. Considering a body weight of approximately 25 g per mouse and a daily drinking volume of approximately 5 mL, the final concentration of the ascorbate solution was 5 g/L (pH 7.0). Mice from the placebo (PLC) cohort received autoclaved tap water only. Of note, the daily inter-individual drinking volumes between cages within and between respective cohorts were comparable. In four individual experiments, n = 5/5/4/4 ascorbate treated mice and n = 6/6/6/6 PLC controls were analyzed.

### *C. jejuni* infection

As previously described, mice were challenged with 10^9^ CFU of the *C. jejuni* strain 81–176 (that had initially been isolated from a diseased patient suffering from bloody diarrhea) in a volume of 0.3 mL phosphate buffered saline (PBS; Gibco, life technologies, UK) on two successive days (days 0 and 1) by oral gavage^15^. In order to avoid contaminations, mice were kept and handled under strict aseptic conditions.

### Evaluation of clinical conditions

The clinical conditions of mice were evaluated daily (starting four days before and lasting until day 6 after *C. jejuni* infection) and quantitated via standardized cumulative clinical scores (maximum 12 points), addressing the abundance of blood in faeces (0: no blood; 2: microscopic detection of blood by the Guajac method using Haemoccult, Beckman Coulter/PCD, Germany; 4: macroscopic blood visible), stool consistency (0: formed faeces; 2: pasty faeces; 4: liquid faeces), and the clinical aspect (0: normal; 2: ruffled fur, less locomotion; 4: isolation, severely compromised locomotion, pre-final aspect) as described earlier^27^.

### Sampling methods

Six days after the infection, mice were sacrificed by inhalation of isoflurane (Abbott, Germany). Luminal gastrointestinal samples (i.e., from colon, ileum, duodenum, and stomach) and *ex vivo* biopsies from intestinal (colon, ileum, MLN) and extra-intestinal (liver, kidneys and lungs) compartments were taken under aseptic conditions. To generate individual serum probes, cardiac blood was collected (approximately 1.0 mL). Intestinal samples for microbiological, immunological and immunohistopathological assays were taken in parallel from each mouse.

### Histopathology

For histopathological analyses, sections (thickness 5 µm) of formalin-fixed (5%) and paraffin-embedded colonic *ex vivo* biopsies were used, stained with hematoxylin and eosin (H&E), and examined by light microscopy (100x magnification). The histopathological changes in the large intestines were quantitatively evaluated following an established histopathological scoring system ranging from 0 to 4 as described earlier^59^. Score 1: intact epithelium with minimal inflammatory cell infiltrates in the mucosa. Score 2: mild hyperplasia and mild goblet cell loss with mild inflammatory cell infiltrates in the mucosa and submucosa. Score 3: moderate goblet cell loss with moderate inflammatory cell infiltrates in the mucosa. Score 4: marked goblet cell loss, multiple crypt abscesses and crypt loss with marked inflammatory cell infiltration into in the mucosa and submucosa.

### Immunohistochemical assays

*In situ* immunohistochemical analyses were conducted as previously reported^87,88^. Briefly, paraffin sections (5 μm) derived from *ex vivo* biopsies of interest (i.e., colon, liver, kidneys, lungs) were stained with primary antibodies directed against cleaved caspase 3 (Asp175, Cell Signaling, Beverly, MA, USA, 1:200) to detect apoptotic epithelial cells; with F4/80 (# 14–4801, clone BM8, eBioscience, San Diego, CA, USA, 1:50) to detect macrophages/monocytes; with CD3 (#N1580, Dako, 1:10) to detect T lymphocytes; and furthermore, with B220 (No. 14-0452-81, eBioscience; 1:200) to detect B lymphocytes. The sections were incubated with the primary antibody for 30 min followed by another 30 min of incubation with the respective secondary antibody (for anti-caspase-3 and anti-CD3 staining: biotinylated donkey anti-rabbit antibody; for anti-F4/80 and anti-B220: biotinylated rabbit anti-rat antibody; all from Dianova, Hamburg, Germany). As detection system, the Streptavidin-Alkaline Phosphatase Kit (Dako) using Fast Red as chromogen was applied. To generate negative controls, the primary antibodies had been excluded. The examination of positively stained cells was undertaken by light microscopy (magnification 100x and 400x). The average number of respective positively stained cells for each mouse was calculated within at least six high power fields (HPF, 0.287 mm^2^, 400x magnification) by a blinded independent investigator. An Axiolmager Z1 microscope was used for the generation of images, which were subsequently processed with the Axiovision software (Carl Zeiss MicroImaging, Jena, Germany).

### The colonization and translocation of *C. jejuni*

The *C. jejuni* loads were quantitatively surveyed in faeces samples taken every days after infection, and further, upon necropsy in gastrointestinal luminal samples (taken from stomach, duodenum, ileum and colon) and in homogenized *ex vivo* biopsies derived from MLN, spleen, liver, kidneys and lungs as well as in cardiac blood by culture as stated earlier^15,89^. The detection limit of viable pathogens was ≈100 CFU per g. For the determination of cumulative translocation rates of viable *C. jejuni* into respective extra-intestinal compartments, the ratio of the sum of culture-positive mice and the total numbers of analyzed animals (in %) out of four experiments were calculated.

### Measurements of intestinal and extra-intestinal pro-inflammatory mediators

Longitudinally sliced and in PBS washed colonic *ex vivo* biopsies (strips of approximately 1 cm^2^) as well as *ex vivo* biopsies derived from liver (approximately 1 cm^3^) and MLN (3–4 lymph nodes) were cultured for 18 h at 37 °C in 24-flat-bottom well culture plates (Nunc, Germany) containing 500 μL serum-free RPMI 1640 medium (Gibco, life technologies, UK) that was supplemented with penicillin (100 U/mL) and streptomycin (100 µg/mL; PAA Laboratories, Germany). Using the Mouse Inflammation Cytometric Bead Assay (CBA; BD Biosciences, Germany) the culture supernatants and serum samples were analyzed for TNF, IFN-γ and IL-6 on a BD FACS Canto II flow cytometer (BD Biosciences). The nitric oxide concentrations were determined by the Griess reaction^85,90^. The pro-inflammatory mediator levels were normalized to the protein concentrations measured in the supernatant of the respective organ homogenate^88^.

### Data analysis

Data from four independently performed experiments were pooled and analyzed. The Mann-Whitney test (GraphPad Prism v7, USA) was used for determination of medians and levels of significance for pairwise comparisons of not normally distributed data, whereas for multiple comparisons, the one-sided ANOVA with Tukey post-correction or the Kruskal-Wallis test with Dunn’s post-correction were used. Two-sided probability (p) values ≤ 0.05 were considered significant.

## Supplementary information

Supplementary Figure 1.

Supplementary Figure 2.

Supplementary Figure 3.

Supplementary Figure 4.

Supplementary Figure 5.

Supplementary Figure 6.

Supplementary Figure 7.
