# Supplementary material for: Vitamin C alleviates acute enterocolitis in Campylobacter jejuni infected mice
Source: Sci Rep. 2020 Feb 19;10:2921. doi: 10.1038/s41598-020-59890-8 (PMC7031283; doi:10.1038/s41598-020-59890-8)
Supplement: Supplementary file 1 — Supplementary Figure 1. [file 41598_2020_59890_MOESM1_ESM.pdf]

Vitamin C alleviates acute enterocolitis in *Campylobacter jejuni* infected mice

**Soraya Mousavi, Ulrike Escher, Elisa Thunhorst, Sophie Kittler, Corinna Kehrenberg,
Stefan Bereswill and Markus M. Heimesaat**

	6	11	22	44	88	176	352	705	1409	2818	5636	MIC_{50}	MIC_{90}
Ascorbic acid pH 7.3	0	0	0	0	0	0	3	11	12	4	0	1409	2818

Determination of minimal inhibitory concentrations of ascorbate

In order to assess the antimicrobial effect of synthetic ascorbate, 20 *C. jejuni* isolates including the reference strain 81-176 used for infection of mice were tested for their minimal inhibitory concentration by the broth microdilution method. Settings of inoculum density, growth medium and conditions as well as incubation time were applied according to the recommendations of the Clinical and Laboratory Standards Institute (CLSI) given in the document VET01-Ed5 [78]. Twofold serial dilutions ranging from 0.03 – 32.0 mmol/L (6 - 5636 µg/mL) for ascorbate were tested. Stock solutions were prepared in Mueller-Hinton broth (Oxoid, Germany) and adjusted to pH 7.3. *C. jejuni* reference strain DSM 4688 was used for quality control purposes. The MIC value of the reference strain was tested in advance in three independent experiments applying both, the broth microdilution and macrodilution method.
