# Supplementary material for: Vitamin C alleviates acute enterocolitis in Campylobacter jejuni infected mice
Source: Sci Rep. 2020 Feb 19;10:2921. doi: 10.1038/s41598-020-59890-8 (PMC7031283; doi:10.1038/s41598-020-59890-8)
Supplement: Supplementary file 2 — Supplementary Figure 2. [file 41598_2020_59890_MOESM2_ESM.pdf]

Vitamin C alleviates acute enterocolitis in *Campylobacter jejuni* infected mice

**Soraya Mousavi, Ulrike Escher, Elisa Thunhorst, Sophie Kittler, Corinna Kehrenberg,
Stefan Bereswill and Markus M. Heimesaat**

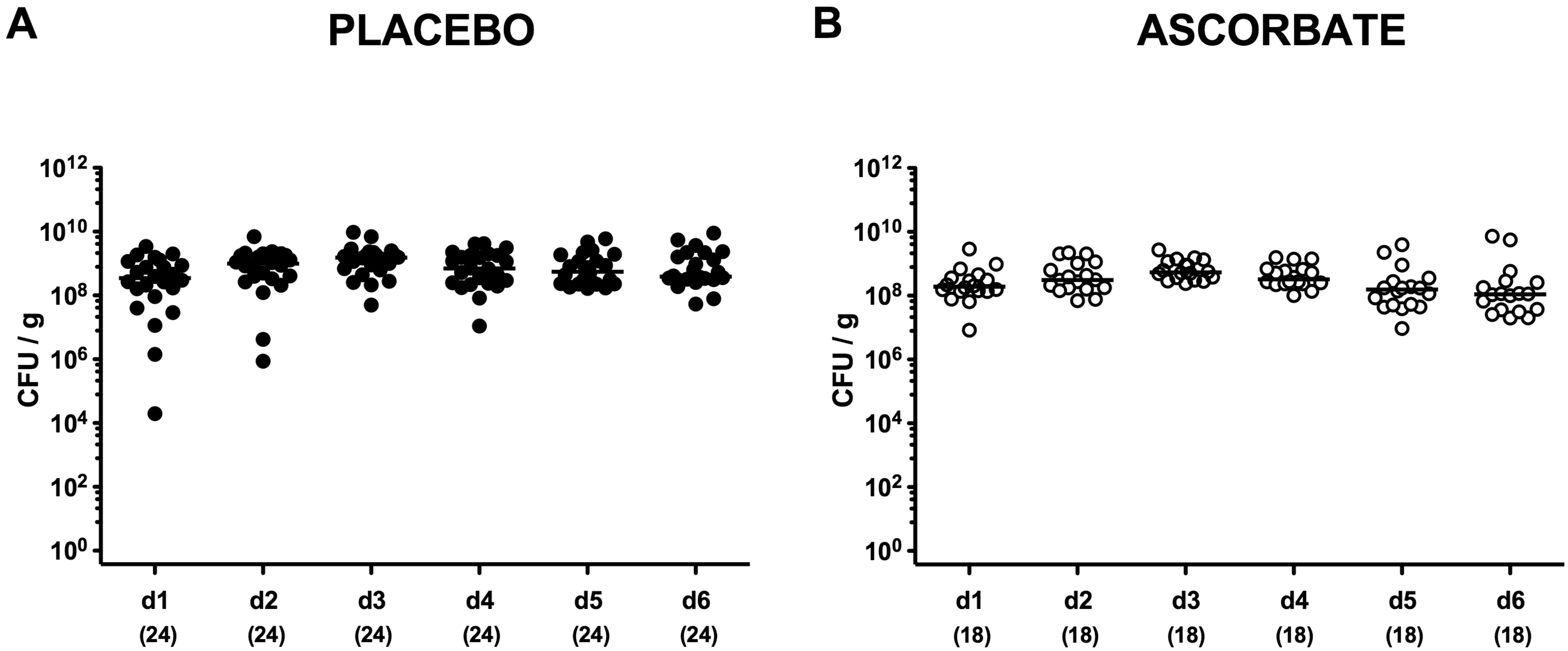

Pathogenic loads over time following ascorbate treatment of *C. jejuni* infected secondary abiotic IL-10$^{-/-}$ mice. Starting four days before peroral *C. jejuni* infection, secondary abiotic IL-10$^{-/-}$ mice were treated with synthetic ascorbate (ASCOR; open circles) or placebo (PLC; filled circles) via the drinking water. Faecal pathogenic loads were assessed on a daily basis post-infection (expressed as colony forming units per gram, CFU / g). Medians, significance levels (p-values; ** $p<0.01$; *** $p<0.001$) assessed by the Mann-Whitney U test (for pairwise comparisons of PLC vs ASCOR at respective time points) and numbers of analyzed animals (in parentheses) are indicated. Data were pooled from four independent experiments.
