# Supplementary material for: Vitamin C alleviates acute enterocolitis in Campylobacter jejuni infected mice
Source: Sci Rep. 2020 Feb 19;10:2921. doi: 10.1038/s41598-020-59890-8 (PMC7031283; doi:10.1038/s41598-020-59890-8)
Supplement: Supplementary file 3 — Supplementary Figure 3. [file 41598_2020_59890_MOESM3_ESM.pdf]

Vitamin C alleviates acute enterocolitis in *Campylobacter jejuni* infected mice

Soraya Mousavi, Ulrike Escher, Elisa Thunhorst, Sophie Kittler, Corinna Kehrenberg, Stefan Bereswill and Markus M. Heimesaat

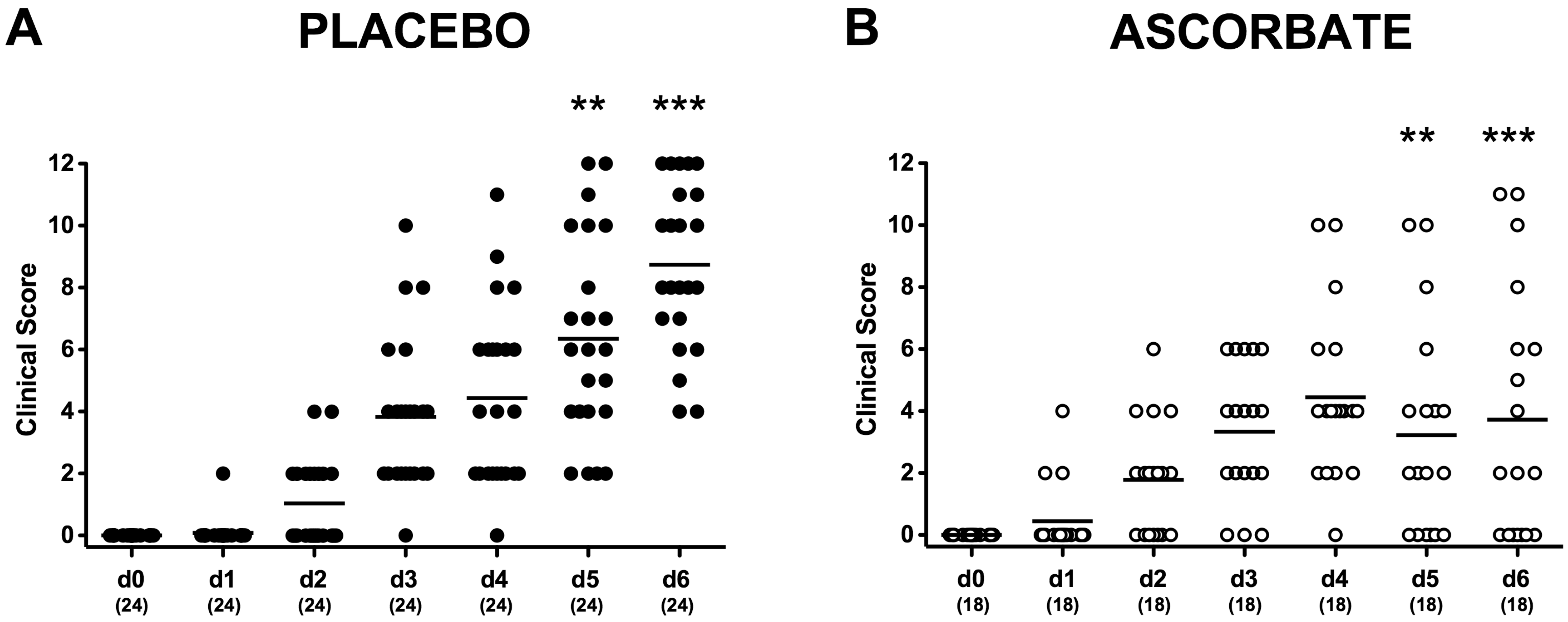

Kinetic survey of clinical conditions in *C. jejuni* infected secondary abiotic IL-10$^{-/-}$ mice. Starting four days prior peroral *C. jejuni* infection, secondary abiotic mice were treated with synthetic ascorbate (open circles) of placebo (closed circles) via the drinking water. Severities of clinical symptoms were surveyed daily from d0 until d6 post-infection applying a standardized clinical scoring system (see methods). Medians, significance levels (p-values, ** $p<0.01$, *** $p<0.001$) assessed by the Mann-Whitney U test (for pairwise comparisons of PLC vs ASCOR at identical time points) and numbers of analyzed animals (in parentheses) are indicated. Data were pooled from four independent experiments.
