# Supplementary material for: Vitamin C alleviates acute enterocolitis in Campylobacter jejuni infected mice
Source: Sci Rep. 2020 Feb 19;10:2921. doi: 10.1038/s41598-020-59890-8 (PMC7031283; doi:10.1038/s41598-020-59890-8)
Supplement: Supplementary file 4 — Supplementary Figure 4. [file 41598_2020_59890_MOESM4_ESM.pdf]

Vitamin C alleviates acute enterocolitis in *Campylobacter jejuni* infected mice

Soraya Mousavi, Ulrike Escher, Elisa Thunhorst, Sophie Kittler, Corinna Kehrenberg, Stefan Bereswill and Markus M. Heimesaat

Representative photomicrographs of immunohistochemically stained colonic paraffin sections taken from ascorbate treated, *C. jejuni* infected mice. Starting four days before peroral *C. jejuni* infection, secondary abiotic IL-10$^{-/-}$ mice were treated with synthetic ascorbate (ASCOR) or placebo (PLC) via the drinking water. Naive mice served as uninfected and untreated controls. Apoptotic colonic epithelial cells (positive for cleaved caspase3) were quantitatively assessed microscopically in immunohistochemically stained large intestinal paraffin sections at day 6 post-infection. Photomicrographs shown are representative for four independent experiments.

Apoptotic Cells (COLON)

Naive

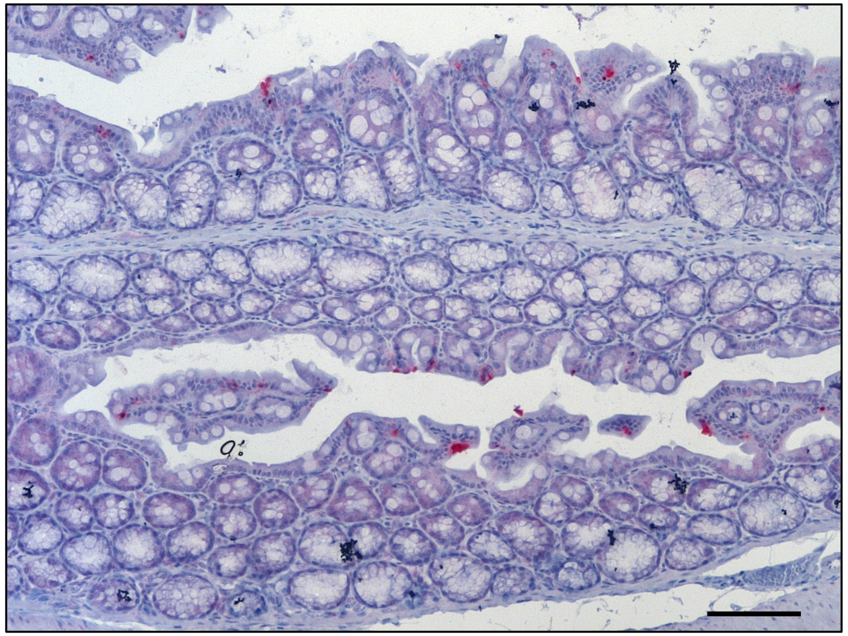

PLC

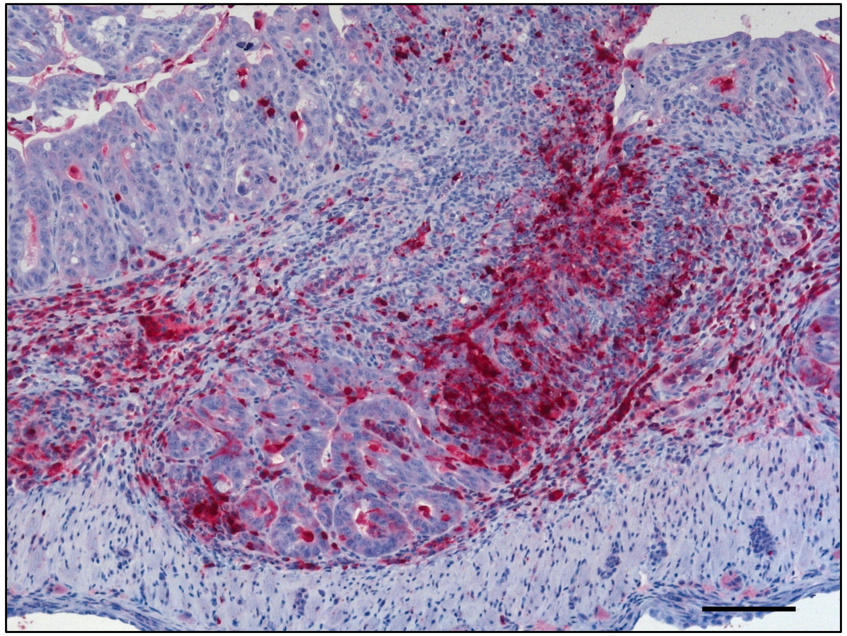

ASCOR

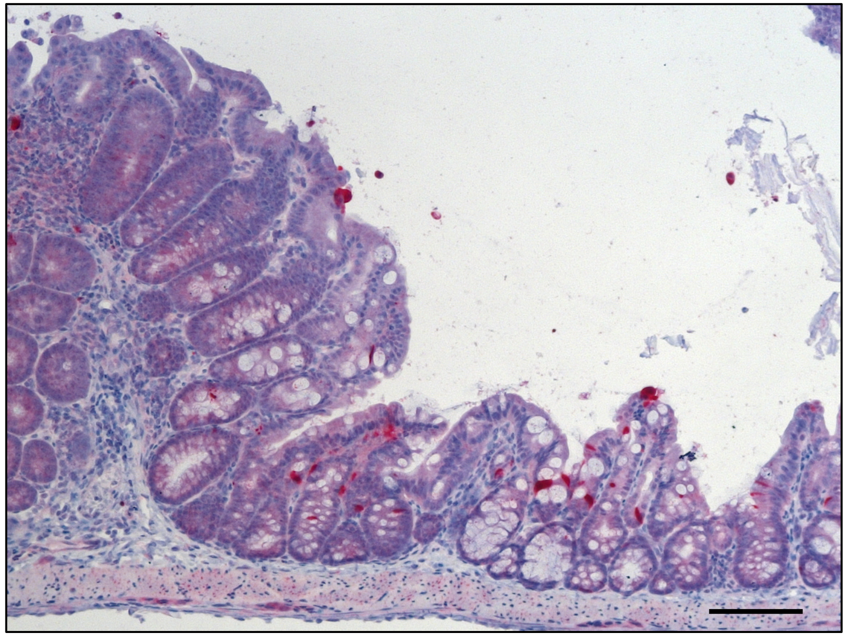

(100 x magnification, scale bar 100 µm)
