# Supplementary material for: Vitamin C alleviates acute enterocolitis in Campylobacter jejuni infected mice
Source: Sci Rep. 2020 Feb 19;10:2921. doi: 10.1038/s41598-020-59890-8 (PMC7031283; doi:10.1038/s41598-020-59890-8)
Supplement: Supplementary file 5 — Supplementary Figure 5. [file 41598_2020_59890_MOESM5_ESM.pdf]

Vitamin C alleviates acute enterocolitis in *Campylobacter jejuni* infected mice

Soraya Mousavi, Ulrike Escher, Elisa Thunhorst, Sophie Kittler, Corinna Kehrenberg, Stefan Bereswill and Markus M. Heimesaat

Representative photomicrographs of immunohistochemically stained colonic paraffin sections taken from ascorbate treated, *C. jejuni* infected mice. Starting four days before peroral *C. jejuni* infection, secondary abiotic IL-$10^{-/-}$ mice were treated with synthetic ascorbate (ASCOR) or placebo (PLC) via the drinking water. Naive mice served as uninfected and untreated controls. **(A)** macrophages and monocytes (positive for F4/80), **(B)** T lymphocytes (positive for CD3), and **(C)** B lymphocytes (positive for B220) in the colonic mucosa and lamina propria were quantitatively assessed microscopically in immunohistochemically stained large intestinal paraffin sections at day 6 post-infection. Photomicrographs shown are representative for four independent experiments.

A **Macrophages / Monocytes (COLON)**

Naive **PLC** **ASCOR**

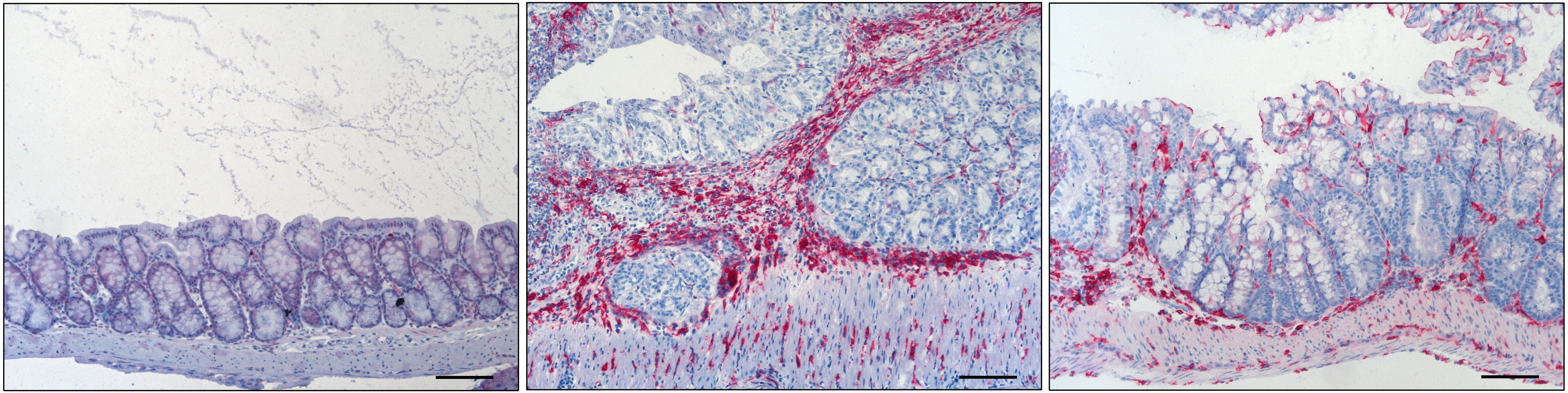

(100 x magnification, scale bar 100 μm)

B

T Lymphocytes (COLON)

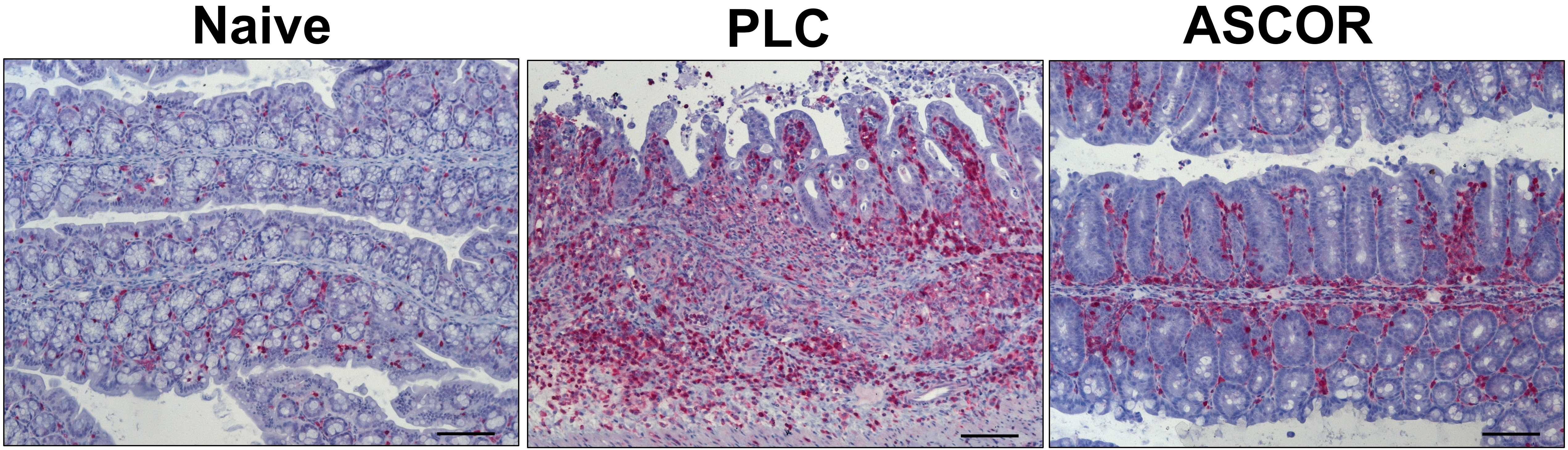

(100 x magnification, scale bar 100 µm)

C

B Lymphocytes (COLON)

Naive PLC ASCOR

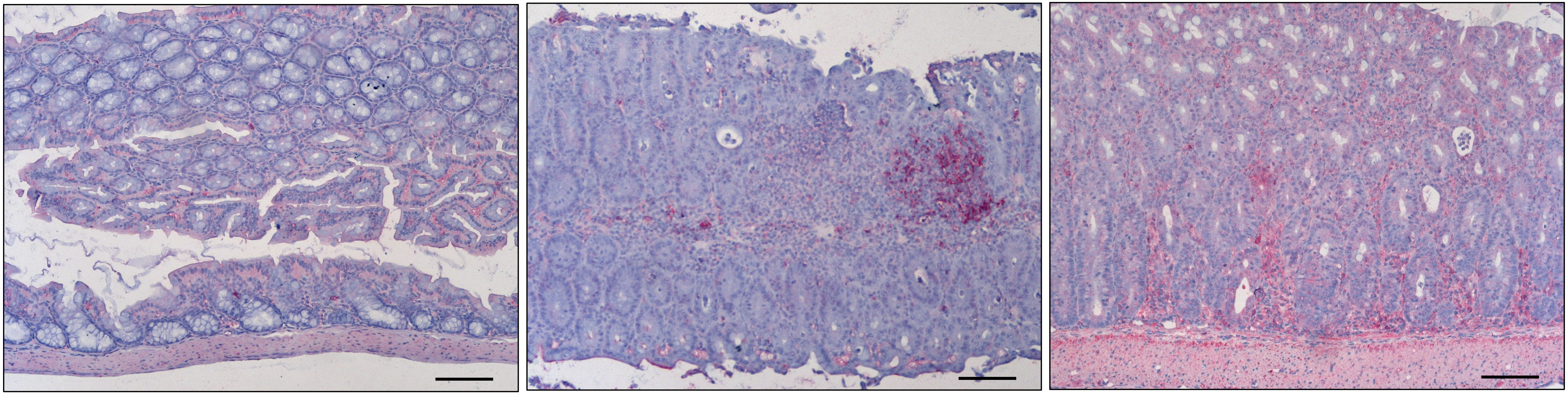

(100 x magnification, scale bar 100 µm)
