# Supplementary material for: Vitamin C alleviates acute enterocolitis in Campylobacter jejuni infected mice
Source: Sci Rep. 2020 Feb 19;10:2921. doi: 10.1038/s41598-020-59890-8 (PMC7031283; doi:10.1038/s41598-020-59890-8)
Supplement: Supplementary file 6 — Supplementary Figure 6. [file 41598_2020_59890_MOESM6_ESM.pdf]

# Vitamin C alleviates acute enterocolitis in *Campylobacter jejuni* infected mice

**Soraya Mousavi, Ulrike Escher, Elisa Thunhorst, Sophie Kittler, Corinna Kehrenberg,
Stefan Bereswill and Markus M. Heimesaat**

**Representative photomicrographs of immunohistoichemically stained extra-intestinal paraffin sections taken from ascorbate treated, *C. jejuni* infected mice.** Starting four days before peroral *C. jejuni* infection, secondary abiotic IL-10$^{-/-}$ mice were treated with synthetic ascorbate (ASCOR) or placebo (PLC) via the drinking water. Naive mice served as uninfected and untreated controls. In **(A)** kidney, **(B)** lung and **(C)** liver apoptotic cells (positive for caspase3) as well as **(D)** hepatic T lymphocytes (positive for CD3) were quantitatively assessed microscopically in immunohistochemically stained paraffin sections taken from respective compartments at day 6 post-infection. Photomicrographs shown are representative for four independent experiments.

# A Apoptotic Cells (KIDNEY)

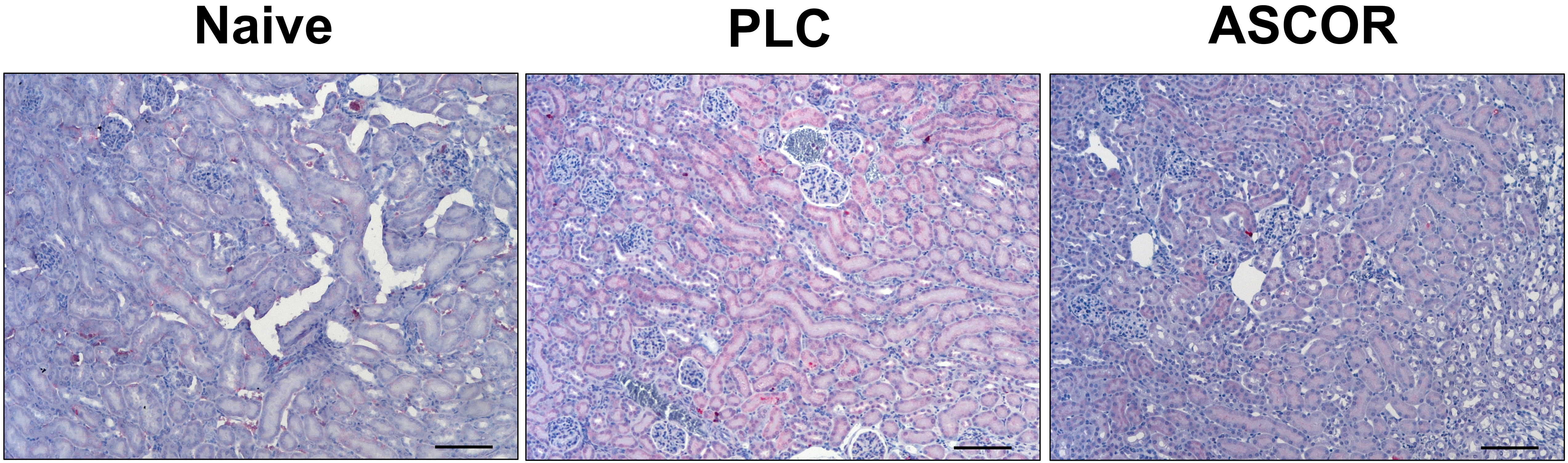


(100 x magnification, scale bar 100 μm)

**B** **Apoptotic Cells (LUNG)**

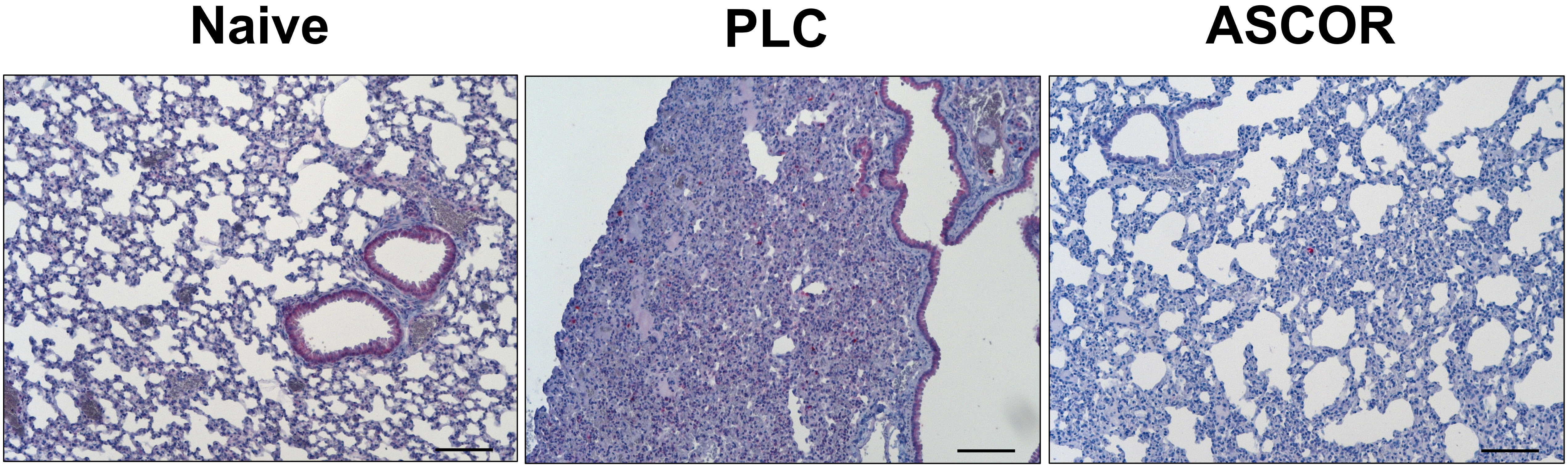


(100 x magnification, scale bar 100 μm)

C

# Apoptotic Cells (LIVER)

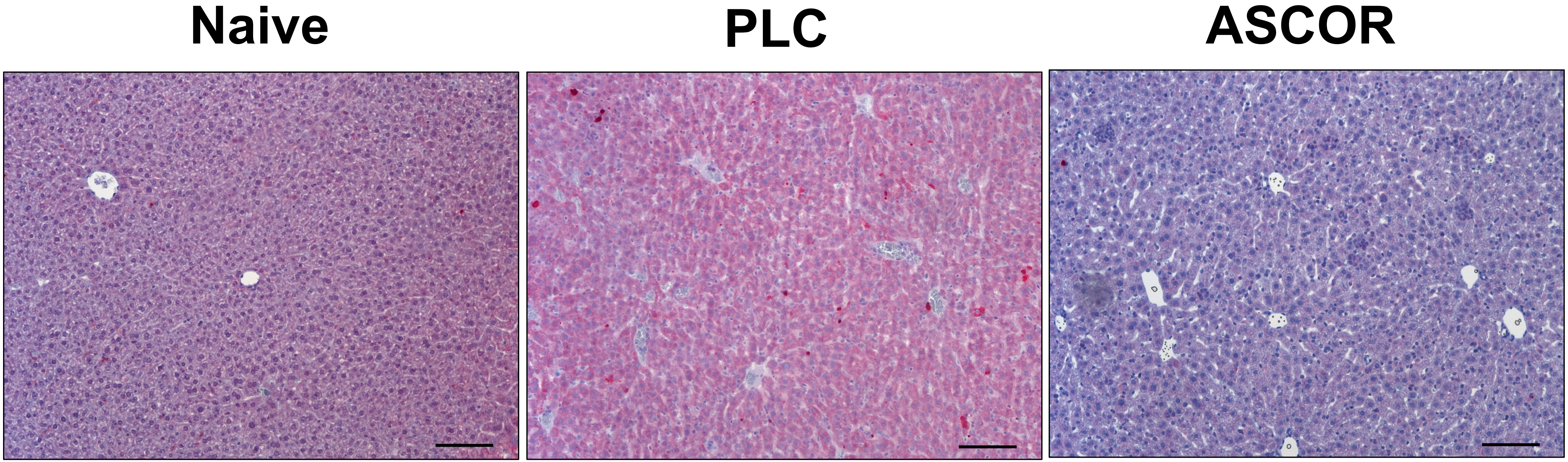


(100 x magnification, scale bar 100 μm)

# D T Lymphocytes (LIVER)

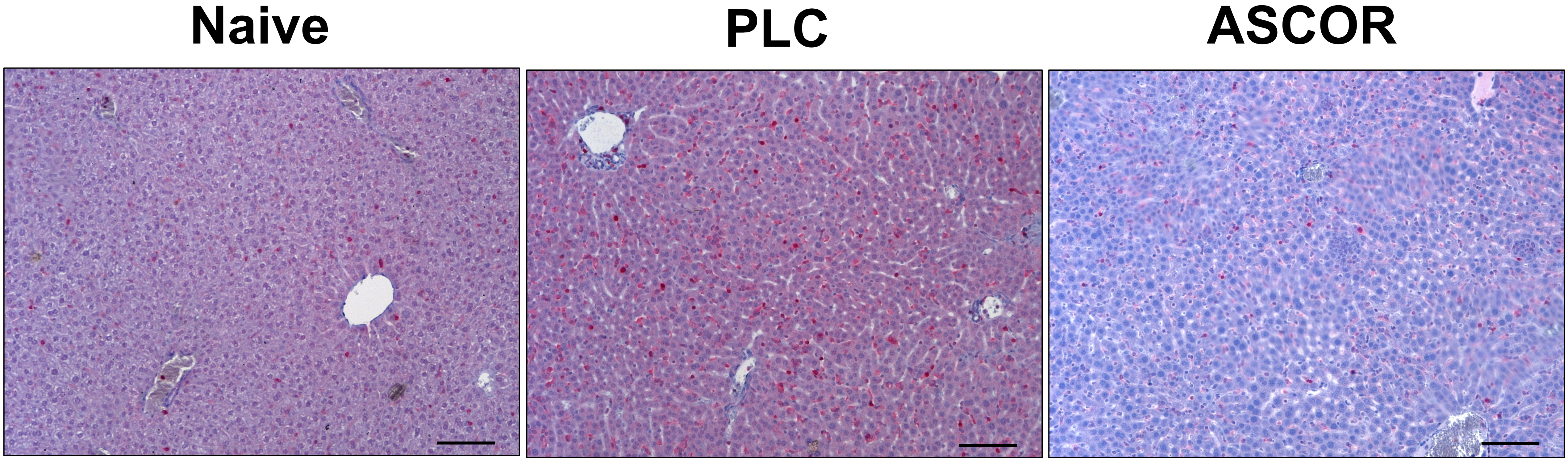


(100 x magnification, scale bar 100 µm)
