# Supplementary material for: Vitamin C alleviates acute enterocolitis in Campylobacter jejuni infected mice
Source: Sci Rep. 2020 Feb 19;10:2921. doi: 10.1038/s41598-020-59890-8 (PMC7031283; doi:10.1038/s41598-020-59890-8)
Supplement: Supplementary file 7 — Supplementary Figure 7. [file 41598_2020_59890_MOESM7_ESM.pdf]

Vitamin C alleviates acute enterocolitis in *Campylobacter jejuni* infected mice

Soraya Mousavi, Ulrike Escher, Elisa Thunhorst, Sophie Kittler, Corinna Kehrenberg, Stefan Bereswill and Markus M. Heimesaat

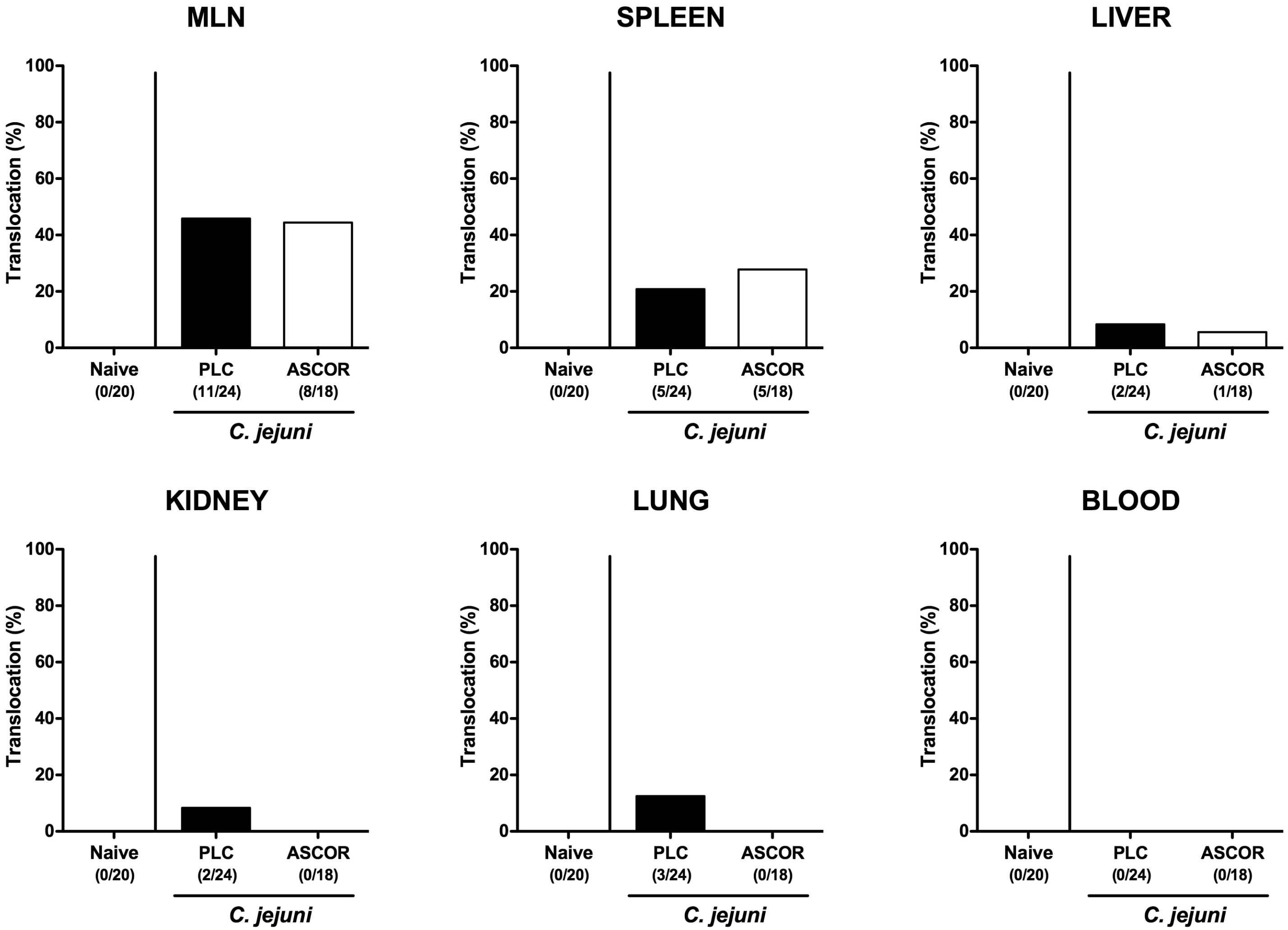

Bacterial translocation in ascorbate treated mice following *C. jejuni* infection. Starting four days before peroral *C. jejuni* infection, secondary abiotic IL-10$^{-/-}$ mice were treated with synthetic ascorbate (ASCOR; open circles) or placebo (PLC; filled circles) via the drinking water. At day 6 post-infection, the abundance of *C. jejuni* was assessed in *ex vivo* biopsies derived from mesenteric lymph nodes (MLN), spleen, liver, kidney, lung, and cardiac blood as indicated by culture. The cumulative relative translocation rates of viable pathogens into the respective compartment as assessed from four independent experiments are indicated (in %).
